# Mesenteric fibromatosis after resection for gastrointestinal stromal tumor of stomach: A case report

**DOI:** 10.1097/MD.0000000000008792

**Published:** 2017-12-01

**Authors:** Yiming Chu, Qingqu Guo, Dan Wu

**Affiliations:** Department of Surgery, The Second Affiliated Hospital, College of Medicine, Zhejiang University, Zhejiang, P.R. China.

**Keywords:** beta-catenin, CD117, gastrointestinal stromal tumor, immunohistochemical staining, mesenteric fibromatosis

## Abstract

**Rationale:**

Gastrointestinal stromal tumor and mesenteric fibromatosis are rare mesenchymal tumors. Coexistence of these two diseases is uncommon, with only a few anecdotal reports of individuals.

**Patient concerns:**

Clinical data and treatment of a 43-year-old man with subsequent mesenteric fibromatosis from gastrointestinal stromal tumor are summarized. The Ethics Committee of The Second Affiliated Hospital, College of Medicine, Zhejiang University approved this study, and the patient provided written informed consent form.

**Diagnoses:**

The initial diagnosis of the recurrent mesenteric mass was recurrent gastrointestinal stromal tumor.

**Interventions:**

The operation was performed as possible at the time when the mass was found after the first surgery.

**Outcomes:**

The diagnosis was revised as mesenteric fibromatosis according to the postoperative immunohistochemical staining. The postoperative condition was normal without adjuvant therapy and no recidivation has been found.

**Lessons:**

The potential for the coexistence of gastrointestinal stromal tumor and mesenteric fibromatosis should always be considered.

## 1 Introduction

Gastrointestinal stromal tumor (GIST) is the most frequent mesenchymal tumor, but it is still rare among all the gastrointestinal tumors.^[1,2]^ GIST frequently shows an unpredictable malignant potential and it tends to recur and leads to distant metastases.^[3]^ Mesenteric fibromatosis is also rare. It lacks metastatic potential but exhibits a high degree of local invasion and recurrence.^[4]^ Distinguishing between these 2 tumors is necessary, since mesenteric fibromatosis may be diagnosed as a recurrence of GIST when it occurs after the resection of GIST. These tumors are often confused clinically, radiologically, and even pathologically.^[3,5,6]^ Immunohistochemical staining may be meaningful to distinguish mesenteric fibromatosis from the recurrence of GIST. We report the case of a patient who suffered mesenteric fibromatosis that developed after resection of GIST.

## 2 Case presentation

**Figure 1:**
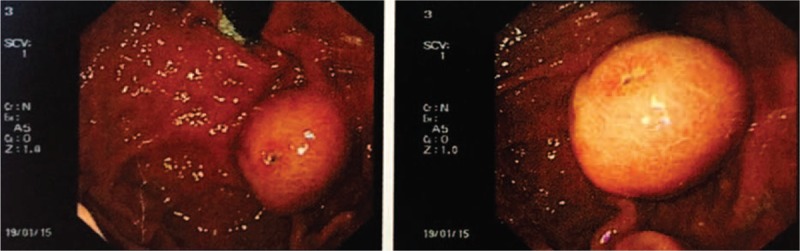
Macroscopic observation of the gastroscopy material revealed a protruding lesion with an ulcer at the top of the mass.

**Figure 2:**
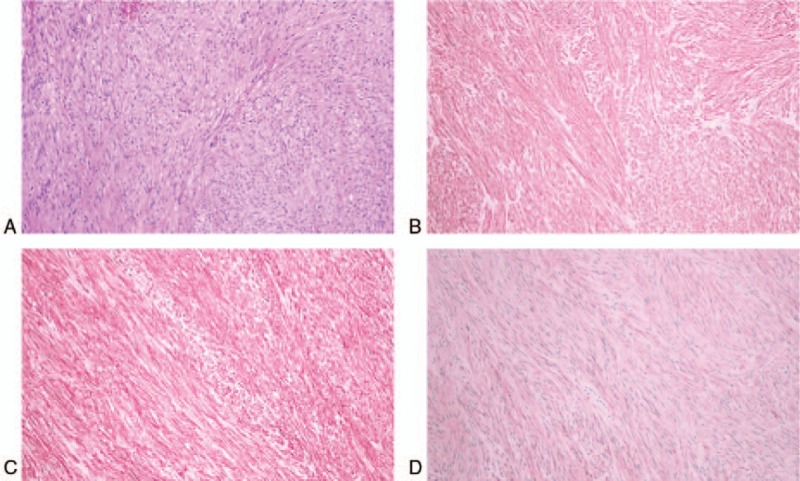
The lesion showed disarray of spindle cells (Hematoxylin and Eosin staining, ×200, A). The tumor expressed c-kit (B), CD34 (C), and DOG-1 (D) on immunohistochemical staining.

A 43-year-old man was referred to our hospital on January 22, 2015 because of epigastric pain and a 30-year history of repeated heartburn that had been worse in the prior decade. He had no previous medical or medication history. Laboratory data and physical examination revealed no abnormalities. Gastroscopy revealed lesions in the fundus of the stomach (Fig. 1). Computed tomography (CT) demonstrated a likely benign tumor. The primary impression was leiomyoma of the stomach. The patient was treated with endoscope submucosal dissection. Pathological examination found disarranged spindle cells (Fig. 2A). Immunohistochemistry staining was positive for cluster of differentiation 117 (CD117) (Fig. 2B), cluster of differentiation 34 (CD34) (Fig. 2C), and discovered on GIST-1 (DOG-1) (Fig. 2D), and negative for smooth muscle actin (SMA), desmin, and S-100. The size of specimen was 3.0 × 2.6 cm. The mitotic rate was 20/50 per high power field. Based on the immunohistochemical examination, the patient was diagnosed as a GIST, which was classified into high risk level with no doubt.^[7]^ Subsequently, surgical excision of the lesions was done. The tumor was removed with combined resection of some portions of the omentum. This patient recovered well postoperatively.

**Figure 3:**
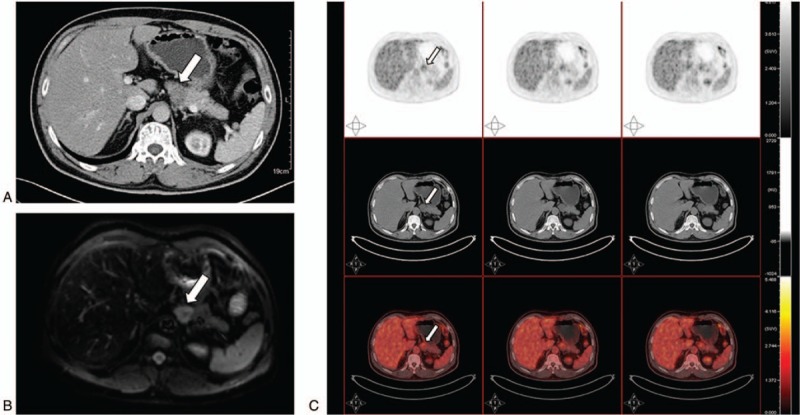
The pancreas was oppressed by the lump located near the fundus of the stomach on contrast-enhanced CT (A, arrow) and the DWI of magnetic resonance image (B, arrow). The tumor could be identified in PET (C, arrow). CT = computed tomography, PET = positron emission tomography.

**Figure 4:**
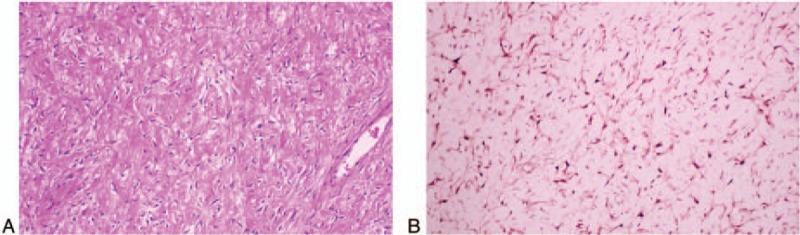
The tumor showed fibroblastic proliferation appearing as bundles of spindle cells (Hematoxylin and Eosin staining, ×200, A). Immunohistochemical staining was positive for nuclear beta-catenin (B).

After the patient was discharged from our hospital, imatinib mesylate was used for only 1 month as adjuvant therapy owing to the severe side-effects. One year and 2 months after the surgery, the physical examination suggested a mass in the mid-abdomen. There were no obvious abnormalities in the laboratory findings. A CT scan identified a lump in the posterior wall of the stomach fundus (Fig. 3A). The mass impinged on the body of the pancreas. Magnetic resonance imaging showed an abnormally enhanced 3.0 × 2.4 cm behind the stomach fundus (Fig. 3B). Positron emission tomography (PET) revealed that the standardized uptake value (SUV max) of the mass was 3.45, and no other abnormal concentration was detected (Fig. 3C). With a presumptive diagnosis as a recurrent GIST, surgery was performed as possible 1.5 years after the first surgery. The tumor was located at the surface of the corpus pancreatis and the boundaries of the mass blurred to obscurity. The tumor was 4.0 × 4.0 cm in size. Unexpectedly, on the omentum majus, another hard texture tubercle was found. It was about 2.0 × 2.0 cm in size. Pathologic examination revealed that both tubercles were strongly positive for SMA, desmin, S-100 as well as nuclear beta-catenin (Fig. 4). On immunohistochemical staining, CD117 was suspiciously positive and CD34 was weakly focally positive, while DOG-1 was definitively negative. Based on these findings, these 2 subsequent tumors were both diagnosed as mesenteric fibromatosis and not recurrent GIST. The postoperative condition was normal without adjuvant therapy and no recidivation has been found until May 4, 2017.

## 3 Discussion

GIST is the most common tumor of mesenchymal tissue in the gastrointestinal tract. The incidence of GIST ranges from 10.0 to 12.4 per million population annually.^[8,9]^ One of the most frequent sites is the stomach. The majority of GIST cases appear as sporadic lesions other than frequently multiple lesions. In addition, GIST is more likely in patients with neurofibromatosis type 1.^[10]^ On immunohistochemical staining, about 90% to 100% of GIST express KIT(CD117) and approximately 70% to 90% are express CD34. Almost 80% of GIST cases have a mutation in KIT (also denoted c-kit) due to the activation of the KIT receptor, and 7% have a mutation in platelet-derived growth factor receptor alpha (PDGFRA).^[11]^ GIST patients tend to show PDGFRA mutation while lacking KIT mutation.^[12]^ Surgical excision is the most common treatment for GIST. Many patients experience relapse. Apart from surgery, tyrosine kinase inhibitor, such as imatinib, is an effective treatment of GIST.^[12]^ Presently, imatinib mesylate was the adjuvant therapy after the operation, because the mitotic rate was 20/50 per high power field, which is indicative of high risk. At 14 months postoperatively, a mass in the mid-abdomen appeared unexpectedly. The tentative diagnose was the recurrence of GIST that was imatinib-resistant. CT and PET revealed only one mass, so a second resection was done after a multidisciplinary team discussion. Strangely, there was another mass on the omentum majus both beta-catenin and KIT were positive on postoperative immunohistochemical staining. The findings indicated that the second tumors represented mesenteric fibromatosis.

Morbidity due to mesenteric fibromatosis increased from 2.10 to 5.36 per million population annually from 1993 to 2013.^[13]^ Mesenteric fibromatosis is a group of fibroblastic or myofibroblastic tissue that develops ordinarily because of surgery or trauma experiences, but which can appear spontaneously. About 20% of patients diagnosed as mesenteric fibromatosis are associated with familial adenomatous polyposis, and a few cases have uncertain reasons that may include radiation exposure.^[6,14]^ Marek et al^[6]^ reported the presence of beta-catenin in mesenteric fibromatosis cases, with up to 75% of the cases being positive for KIT. Several studies have reported the absence of CD34. The present case was weakly focally positive for CD34. The dichotomy is confusing and unresolved. Treatment of mesenteric fibromatosis should be individualized and multimodal. Most of the lesions should be resected when the tumor is located and do not violate the root of the mesentery. There may be some complications like short bowel syndrome after aggressive surgery. Lesions that are unable to be removed may be treated with cytotoxic chemotherapy or radiotherapy. The most common chemotherapeutic is doxorubicin.^[15]^ Imatinib mesylate is a target-based molecule that is effective for both GIST and mesenteric fibromatosis that express *c-kit*.^[7,16,17]^

The morbidity of GIST and mesenteric fibromatosis are both rare.^[5,7,18,19]^ In the majority of cases, mesenteric fibromatosis occurred after the resection of GIST.^[14,20]^ Thway et al^[21]^ reported a case of intra-abdominal desmoid tumor after resection for GIST of the sigmoid mesocolon. However, the relevance between these 2 diseases is still unclear. Additionally, there might be a gene mutation leading to genetic predisposition. The incidence of other neoplasms, such as mesenteric fibromatosis, in GISTs is much higher and the mitotic rate increases in patients who suffer from a second primary tumor compared with those only suffer from GIST (*P* = .006).^[22]^ If surgical trauma is the main reason, however, the incidence of mesenteric fibromatosis after resection of the GIST is much higher than the patients who receive another abdominal operation.^[14]^

The discrimination between the recurrence of GIST and mesenteric fibromatosis is difficult since a biopsy cannot be performed. Immunohistochemical staining should be done and the diagnosis should be made along with clinical findings. Beta-catenin, CD34, KIT, and DOG-1 are markers that can discriminate GIST from mesenteric fibromatosis. However, it is essential to distinguish false positive and false negative results, which will lead a misdiagnosis and inappropriate treatments. Nevertheless, complete resection of the mass does make sense for GIST and mesenteric fibromatosis. In recent years, imatinib mesylate has been accepted as an adjuvant chemotherapy for patients with the tumors who do not have the indications for resection, and for whom surgery is too risky an alternative. Sunitinib is effective for GIST, and sorafenib as well as non-steroidal anti-inflammatory drugs are valid for mesenteric fibromatosis.^[23,24]^

## 4 Conclusions

The rare instance as reported in this case highlights that coexistence of GIST and mesenteric fibromatosis should be considered. Exact planning of the follow-up is necessary. More data are needed for decisive and better treatments.

## Acknowledgments

The authors are very grateful to the patient for his consent.
